# Long-Term Safety of Anti-COVID-19 mRNA Vaccines in Patients with Systemic Lupus Erythematosus and Lupus-like Diseases with a Previous History of Myocarditis

**DOI:** 10.3390/microorganisms13102266

**Published:** 2025-09-26

**Authors:** Giovanni Benanti, Marta Secci, Andrea Villatore, Sara Angiulli, Chiara Calabrese, Gabriele Domenico Gallina, Veronica Batani, Giacomo De Luca, Corrado Campochiaro, Giuseppe Pizzetti, Giovanni Peretto, Simone Sala, Enrica P. Bozzolo, Luca Moroni, Marco Matucci-Cerinic, Giuseppe A. Ramirez, Lorenzo Dagna

**Affiliations:** 1 Unit of Immunology, Rheumatology, Rheumatology, Allergy and Rare Diseases, IRCCS San Raffaele Scientific Institute, Via Olgettina 60, 20132 Milan, Italy; benanti.giovanni@hsr.it (G.B.);; 2 Faculty of Medicine, University of Cagliari, Strada Provinciale 8, 09042 Monserrato, Italy; 3 Faculty of Medicine, Vita-Salute San Raffaele University, Via Olgettina 58, 20132 Milan, Italy; 4 Multidisciplinary Disease Unit for Myocarditis and Arrhythmogenic Cardiomyopathies, IRCCS San Raffaele Scientific Institute, Via Olgettina 60, 20132 Milan, Italy; 5 Unit of Cardiology, IRCCS San Raffaele Scientific Institute, Via Olgettina 60, 20132 Milan, Italy

**Keywords:** anti SARS-CoV-2 vaccines, systemic lupus erythematosus, long term safety, myocarditis

## Abstract

Non-viral myocarditis is rare but relatively more frequent in patients with systemic autoimmune diseases (such as systemic lupus erythematosus, SLE, and allied conditions) than in the general population. In rare cases, mRNA-based vaccines can also trigger non-viral myocarditis. Limited data are available about the cardiac safety of mRNA vaccines in this subset of patients. Here, we report data from a third-level hospital on long-term safety, leveraging on a previously described cohort of 13 consecutive patients with SLE, Undifferentiated (UCTD) and Mixed Connective Tissue disease (MCTD), and a history of myocarditis, who had received anti-COVID-19 vaccination between April 2021 and January 2022. Demographics and clinical data (including validated clinometric for SLE) were collected at baseline, at the first available visit following the primary vaccination cycle, after an additional 12 months, and at the last available follow-up after at least 36 months. Twelve patients, seven females, ten with SLE, one MCTD, and one UCTD, had a median follow-up of 41 (35–45) months. One patient was lost at follow-up. No disease flare or sign of myocarditis recurrence were observed. At last visit, all patients were in a low disease activity state (LLDAS), and all but one were in remission, according to the Definition of Remission in SLE (DORIS) criteria. No significant variations in disease activity or damage accrual nor in markers of inflammation and myocardial injury were observed. Our data suggest that mRNA-based anti-COVID-19 vaccines in patients with previous autoimmune myocarditis in the context of SLE and allied conditions have a good long-term safety profile.

## 1. Introduction

Systemic lupus erythematosus (SLE) is an autoimmune connective tissue disease (CTD) with a wide spectrum of clinical presentations, multi-organ involvement, and a relapsing–remitting course. It is widely acknowledged that viral triggers, such as severe acute respiratory syndrome coronavirus 2 (SARS-CoV-2), may unmask latent autoimmune diseases, including SLE, and trigger disease flares [1,2,3]. Previous studies [4] suggest that up to 25% of SLE flares can be preceded by infections. Infection-related flares might exhibit distinct clinical and serological features and often present more severely compared to flares not related to infections [4]. Furthermore, patients with SLE have been reported to experience more severe clinical manifestations of SARS-CoV-2-related disease (COVID-19), with a higher mortality rate compared to the general population [5]. Thus, infection prevention via vaccination is a key objective for the management of patients with CTD [6].

The advent of mRNA-based and viral-vectored anti-COVID-19 vaccines enabled an effective and safe shielding of vulnerable subjects with immune-mediated disorders, including patients with SLE and allied conditions [7,8,9]. Nonetheless, concerns about vaccine safety were raised due to the exclusion of patients with pre-existing alterations of the immune response from vaccine registration trials and after reports of post-vaccine immune-mediated adverse events. Among them, myocarditis received enhanced attention due to its potential severity and its clinical/pathophysiological profile, lying at the crossroads among potential complications of viral infections, vaccine-related inflammatory adverse events, and autoimmunity [10]. Susceptible individuals might in fact develop uncontrolled cardiac injury, possibly triggered by viral infections and maintained and/or amplified by aberrant immune responses [11].

Compared to the baseline risk of myocarditis in the general population and to the risk of vaccine-related myocarditis, the chances of developing myocarditis are higher in patients with COVID-19, especially in the absence of previous vaccination [10,12]. Conversely, vaccine-related myocarditis is associated with a more favourable outcome, including a lower need for mechanical circulatory support and a reduced incidence of cardiogenic shock [11,13]. Vaccine-related myocarditis has predominantly been reported in young individuals between 12 and 39 years, with approximately 80.5% of cases occurring in males. It was most frequently linked to mRNA vaccines, particularly the Moderna vaccine [14]. Myocarditis constitutes an elusive, though clinically relevant, manifestation of SLE and other connective tissue disorders [15]. Epidemiological studies suggest that up to 10% of patients with SLE may develop myocarditis during the course of the disease [14,16]. Compared with other forms of autoimmune myocarditis, SLE-associated myocarditis is characterised by a higher prevalence of depressed left ventricular function and conduction abnormalities, despite potential subclinical presentations [10].

Short-term analyses conducted both in the general population [12,13,17] and in patients with rheumatic disorders [18] yielded reassuring results and consolidated existing knowledge on general vaccine safety in patients with a history of myocarditis, especially in the context of SLE and lupus-like CTDs [19,20,21,22]. To date, however, limited data are known about the long-term cardiac outcomes, data that could further support rheumatologists in the informed management of these patients. Here, building on a previously described, well-characterised cohort of subjects with SLE and lupus-like CTDs with a history of myocarditis, we propose to retrospectively explore possible adverse effects in terms of disease flare (both of myocarditis or of the underlying CTDs) and laboratory parameters over a period of more than three years after receiving the first mRNA-based anti-COVID-19 vaccine dose.

## 2. Materials and Methods

### 2.1. Study Design and Aim of This Study

This is a retrospective observational explorative study. We previously described a prospective observational cohort of 13 patients affected by SLE, MCTD, or UCTD, with a history of non-viral myocarditis [18], consecutively enrolled between April 2021 and January 2022 during the anti-COVID-19 vaccination campaign. In the present study, we performed an exploratory analysis to assess the long-term safety of anti-COVID-19 vaccination, conducting a retrospective evaluation of patients from the same cohort who received at least three years of follow-up from the first vaccine dose. The long-term safety of anti-COVID-19 mRNA vaccines in patients with SLE and SLE allied conditions with a history of previous non-viral myocarditis was assessed considering (1) the occurrence of new episodes of myocarditis; (2) significant variation in underlying disease activity; (3) significant variation in myocardial and disease-specific biomarkers.

### 2.2. Patients

As previously reported [18], among a large population of patients regularly followed in the setting of the Lupus and Connective Tissue Diseases Clinic of San Raffaele University Hospital, a large, third level referral centre in Milan, Italy, we identified patients fulfilling the following criteria: (1) age ≥ 18 years; (2) diagnosis of (a) SLE according to the 2019 American College of Rheumatology (ACR)/European League Against Rheumatism (EULAR) and/or the 2012 SLE International Collaborating Clinics (SLICC) classification criteria for SLE [23]; (b) UCTD as per the 1999 criteria [24]; or (c) MCTD as per Tanaka et al. [25]; (3) eligibility to receive mRNA-based anti-COVID-19 vaccination according to the national vaccination protocol [26]; (4) a history of virus-negative myocarditis proven by endomyocardial biopsy or typical features at magnetic resonance imaging (MRI) [27]. The only exclusion criteria were age < 18 years or refusal to sign informed consent. The enrolment took place between April 2021 and January 2022 during routine scheduled visits. Thus, 11/281 patients with SLE, 1/68 patients with UCTD, and 1/9 patients with MCTD were enrolled. No patients refused to be included in this study. As of April 2025, we retrospectively collected clinical information from the records of patients in the original cohort who had continued regular follow-up visits for at least three additional years after their first vaccine dose. Written informed consent was obtained from all participants under the Panimmuno Research protocol, conforming to the Declaration of Helsinki and approved on 8 March 2018 by the San Raffaele Institutional Review Board, with reference code 22/INT/2018.

### 2.3. Timepoints

Patients enrolled in this study were followed as per routine clinical practice and data were retrieved retrospectively from clinical charts redacted for the usual outpatient management of patients. In this setting, patients were seen on average every 3–6 months according to clinical needs. In addition, due to the lack of standardised procedures for monitoring cardiac function in anti-COVID-19 vaccinees, patients were prescribed to undergo extra laboratory tests ideally within three–five days from the first and second vaccine doses. These laboratory tests included blood cell counts, erythrocyte sedimentation rate, ESR, C-reactive protein, CRP, troponin T, and pro-brain natriuretic peptide, proBNP. The timing and content of these tests were agreed upon by consensus among experienced rheumatologists and cardiologists (GAR, GDL, SS, and GPe) based on available evidence at the time of enrolment, which showed that most vaccine-related adverse events occurred within less than one week from the receipt of a vaccine dose [28,29]. Patients showing abnormal cardiac marker values were invited to undergo tighter serial monitoring according to clinical judgement.

**Figure 1:**
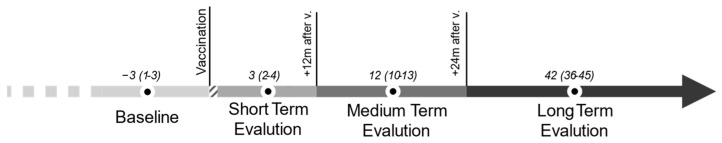
Timepoints of patients’ evaluation. The median (interquartile range) times separating each timepoint from the first vaccination cycle are reported in italic font. v.—vaccination.

We defined the closest patient visit before the first dose of anti-COVID-19 vaccines as the baseline timepoint (Figure 1). The first visit after completion of a full cycle of the first two vaccine doses was categorised as a short-term evaluation timepoint. Assessment of patient status 12 months after the primary vaccination cycle was defined as a medium-term evaluation timepoint. The long-term evaluation timepoint coincided with the last available visit and had to be performed at least 24 months after the short-term assessment.

### 2.4. Connective Tissue Disease Clinical Features

Baseline clinical characteristics recorded in this study included demographics and CTD-related manifestations in patients’ general history. Measures of disease activity, remission, and damage accrual were obtained at each timepoint (see Section 2.3), along with laboratory data and treatment changes. Disease activity was quantitated through the SLE disease activity index 2000 (SLEDAI-2K) and the British Isles Lupus Assessment Group (BILAG) 2004 scale [30]. Damage was estimated through the SLICC/ACR damage index (SDI) [31]. Remission was defined according to the Lupus Low Disease Activity Status (LLDAS) [32] and the Definition of Remission in SLE (DORIS) criteria [33]. We defined an SLE flare when relapse or new manifestations of the disease led to treatment escalation [34].

In addition to myocarditis-specific biomarkers, laboratory data included blood cell counts, markers of renal and liver function, complement C3 and C4, anti-double-stranded DNA antibody (ADNA) titres, CRP levels, and ESR.

Treatment information included the number of patients taking hydroxychloroquine, biotechnological immunomodulants (belimumab), and conventional or biotechnological immunosuppressants. We also recorded the number of patients taking corticosteroids and the dosage of corticosteroid treatments in prednisone equivalents.

### 2.5. Myocarditis Features

Myocardial activity was monitored by an assessment of clinical features, biomarkers, and imaging studies. Markers of cardiac injury and/or overload (troponin T, proBNP) were measured after each dose of the primary cycle of vaccination, at each following timepoint (see Section 2.3) and in the case of symptom exacerbation. Similarly, imaging was performed according to routine clinical indications and anticipated or repeated in the case of symptoms suggesting myocarditis recrudescence. Typical clinical symptoms encompassed chest pain or discomfort, fatigue, dyspnoea, palpitations, and/or syncope. Before vaccination, patients were instructed to report the potential occurrence of these symptoms after each vaccine dose. A myocarditis flare was defined as the occurrence of typical symptoms associated with elevated markers of myocardial necrosis and/or suggestive abnormalities on echocardiography or electrocardiogram (ECG) [28,35]. We also collected data on new cardiovascular events taking place during the follow-up period, including ischaemic heart disease and arrhythmic events.

### 2.6. Statistical Analysis

Statistical analyses were conducted with STATACORP Stata^®^ version 18. The normal distribution of continuous variables was assessed with the Shapiro–Wilk normality test. Continuous variables were expressed as mean and standard deviation, or as the median and interquartile range (IQR) of 25th to 75th percentiles, depending on the distribution of data. Variations in quantitative variables over time were compared intra-individually through the Wilcoxon signed-rank test for repeated measurements. Categorical variables were reported as numbers and percentages and were compared among groups by using Pearson’s Chi-square test. A *p*-value was considered significant when *p* < 0.050, with confidence intervals set at 95%.

## 3. Results

### 3.1. Baseline Characteristics

Of the thirteen patients with baseline data, twelve (92%; seven women and five men) had a long-term follow-up (Table 1). One patient moved to another region and could not be investigated for long-term data. Ten (83%) patients had SLE, one (8%) had MCTD, and one (8%) had UCTD. The median age at the onset of CTD was 30 (25–45) years, and myocarditis occurred after a median of 4 (1–12) years from CTD diagnosis.

At baseline, the majority of patients were either in clinical remission or had low disease activity (Table 1). In SLE patients, 9/10 met LLDAS criteria. The median SLEDAI-2k was 2 (0–2), with only one patient with a score higher than 4. The other subjects were considered in remission by the attending physician. No patients had active myocarditis. The most frequent SLE disease domains according to the BILAG score were musculoskeletal (9; 75%), haematological (8; 66%), and mucocutaneous (8; 66%). Five (41%) had a history of renal involvement (Table 1). At the time of vaccination, two patients were taking low-dose corticosteroid therapy, six (50%) hydroxychloroquine, five (41%) mycophenolate mofetil, two (16%) subcutaneous belimumab, and one (8%) azathioprine. Only two patients were taking corticosteroids. The median daily prednisone dose was 3.1 (2.8–3.4) mg/day. As previously described, no patient underwent treatment changes during vaccination, and all patients received mRNA-based vaccines. Particularly, 11/12 patients received the Pfizer vaccine, and 1/12 received the Moderna vaccine. Two patients had developed a confirmed COVID-19 infection one year prior to vaccination, one 12 months prior to the first dose, the another 7 months before the first dose. Both had a mild infection not requiring hospitalisation and both, according to the national protocol at the time, received a complete vaccination cycle.

### 3.2. Variations in Clinical and Laboratory Features over Time

The primary vaccination cycle occurred within 3 (1–3) months from baseline. Short-, intermediate-, and long-term evaluations were performed 3 (2–4), 12 (10–13), and 42 (36–45) months after the primary vaccination cycle, respectively.

#### 3.2.1. Lupus Activity and Damage Accrual

As previously reported [18], no significant changes in disease activity or damage accrual were observed at short- and medium-term timepoints. At long-term evaluation, all patients were clinically stable and did not require treatment escalation. Considering patients classified with SLE, nine met the LLDAS and DORIS criteria for remission. One could not be attributed to DORIS remission due to mild thrombocytopenia. Consistent with this, the median SLEDAI-2k was 0 (0–2), and no patient had A- or B-grade activity in any BILAG domain. Three patients had C-grade activity in the haematological domain, in one case due to mild leukopenia, in one case due to lymphopenia, and in one case due to mild thrombocytopenia.

Longitudinally, SLEDAI-2k scores decreased significantly from the short- to the long-term evaluation [2 (0–4) to 0 (0–2); *p* = 0.031]. No other significant variations were observed in terms of measures of activity, damage, and remission across each distinct timepoint (Table 2). No increase in therapy was needed during follow-up. Of note, no patient was taking corticosteroids at the last visit, and two patients discontinued mycophenolate mofetil due to prolonged remission.

Comparing baseline laboratory findings [18] with long-term evaluation, no significant variation in blood cell counts or serological markers was observed, except for a slight increase in complement fraction C4 [0.17 (0.13–0.20) vs. 0.23 (0.20–0.30) mg/L *p* = 0.025; Supplemental Table S1].

#### 3.2.2. Clinical and Laboratory Markers of Myocardial Injury

No myocarditis relapse was observed in the short-, medium-, and long-term. In addition, no patient developed myocardial infarction or arrhythmia. Long-term heart ultrasonographic data were available for 9/12 patients, who were re-assessed 35 (29–41) months after the primary vaccination cycle. None of them had signs of active myocarditis or of additional damage compared to baseline evaluation. The median ejection fraction was 60 (55–65)%, and no significant worsening in myocardial function was reported. One patient underwent myocardial MRI, showing no active inflammation of the heart.

**Figure 2:**
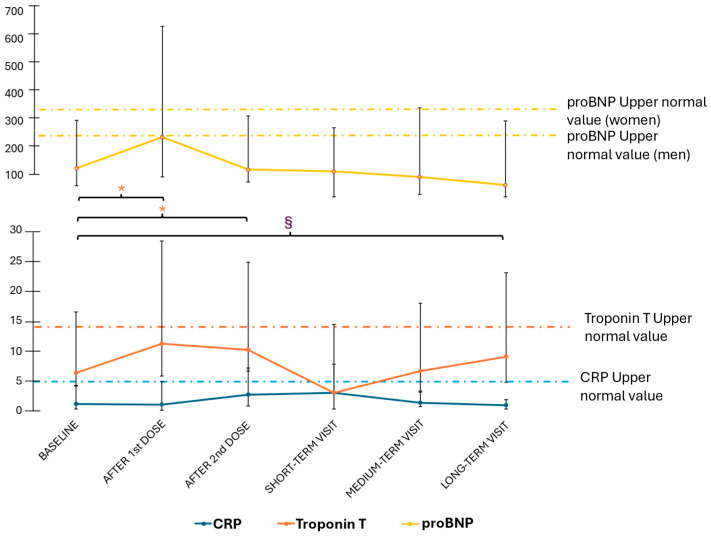
Variations in troponin T (ng/mL; orange), proBNP (pg/mL, yellow), and CRP (g/L; blue) levels at multiple timepoints. Dotted lines in the corresponding colour represent upper normal levels for each test. A statistically but not clinically significant transient increase in troponin T levels was recorded after the first and second dose, while long-term timepoint levels were consistent with baseline. CRP levels at long-term visit showed a slight but significant reduction compared with baseline. Significance levels for *p* < 0.05 are marked with * for troponin levels and § for CRP levels. CRP—C-reactive protein; proBNP—pro B natriuretic peptide.

As previously reported [18], a transient and non-clinically significant rise in troponin T levels was observed following doses 1 and 2 of the primary vaccination cycle, mirrored by a statistically and clinically non-significant increase in proBNP levels. Both progressively showed a tendency towards normalisation. Long-term findings confirmed these trends. Specifically, the median troponin levels were 5.1 ng/mL (2–10.2) at baseline and 8.1 ng/mL (4.2–14) at long-term follow-up (*p* = 0.901), while proBNP levels were 119 pg/mL (53–167) at baseline and 66.5 pg/mL (43.3–232.3) at long-term follow-up (*p* = 0.250; Figure 2). Of note, only two patients (both with SLE) showed abnormal troponin T levels at long-term follow-up, in the context of already abnormal values at baseline. One patient with MCTD had transiently abnormal troponin T levels, which eventually returned within the normal range. With regard to other laboratory findings, we also observed a slight but significant decrease in CRP levels between the baseline and long-term evaluation [1.05 (0.93–3.88) vs. 0.90 (0.50–1.00) g/L, *p* = 0.023]. No difference in biomarker level was observed based on patients’ autoantibody profiles.

## 4. Discussion

This study analysed the long-term clinical trajectories of a group of patients with a history of virus-negative myocarditis due to SLE and lupus-like CTDs after more than 2 years from exposure to a primary immunisation cycle with mRNA anti-COVID-19 vaccines. Adding to the preliminary safety data obtained in the short- and medium-term follow-up of these patients, our results show that the same patients remained clinically stable over time, with regard to both cardiac and non-cardiac manifestations. Imaging and laboratory data confirmed the absence of heart damage progression in the long term. Taken together, these results suggest that mRNA-based vaccines do not confer an increased risk of myocarditis and other cardiac events to patients with an autoimmune background and pre-existing defective immunological tolerance against the myocardium in the short and long term.

In line with our findings, growing evidence indicates that exposure to anti-COVID-19 vaccines is not associated with excess rates of short- and long-term morbidity in patients with immune-mediated diseases [36,37,38]. Conversely, disproportionate rates of autoimmune disease flares along with new-onset autoimmunity and accrual of general morbidity have been observed in association with COVID-19 [39,40,41]. Indeed, infections, in general, constitute an important source of morbidity and mortality in patients with autoimmune systemic diseases, both in terms of infection severity and of enhanced risk of disease flare [42,43].

To combat the additive risk of morbidity conferred by infections, prophylactic measures, including vaccines, are strongly encouraged and generally preferred over infection treatment [6,44] in the management of patients with autoimmune diseases. Furthermore, a higher number of boosts or the use of adjuvanted preparations are often needed to overcome coexisting inborn errors of immunity and/or the detrimental effects of immunosuppression [45,46,47,48,49]. However, vaccination rates in patients with autoimmune diseases remain suboptimal, often due to concerns about vaccination safety by both clinicians and patients [50,51]. The recent COVID-19 pandemic exacerbated this concern, possibly due to the coexistence of rising trends in a lack of trust towards academic institutions and state-driven programmes, defective shielding from fake news, and relatively limited data at the time of decision-making due to the emergency context [52,53,54,55].

Vaccine-related myocarditis constitutes a topic of particular interest in the rheumatological community, as virus-negative myocarditis is a shared, potentially life-threatening complication in multiple immune-mediated disorders [15,56,57]. Consistent with previous evidence in the literature [58], we found that the prevalence of clinically overt autoimmune myocarditis was relatively low in cohorts of SLE and lupus-like CTDs. Furthermore, no association was found between the risk of myocarditis relapse and exposure to mRNA anti-COVID-19 vaccines, both in the short [13,18,59,60] and long term. We also did not detect clear signals of cardiac complications other than myocarditis relapse in our cohort, further corroborating the concept of clinical safety associated with the use of mRNA vaccines in patients with previous myocarditis and autoimmune background and aligning with the existing literature, where relatively low risks of long-term cardiac sequelae are reported even in patients with confirmed vaccine-related myocarditis [61]. This evidence further supports existing position statements by cardiological societies, highlighting the higher risk of short- and long-term cardiac complications due to SARS-CoV-2 than to the anti-COVID-19 vaccine [62,63].

The stability of laboratory and imaging markers of cardiac injury in the long-term follow-up further consolidated our clinical findings. Nonetheless, statistically (but not clinically) significant alterations of troponin T were observed after the first vaccine dose compared to baseline. This evidence conforms with the few other studies reporting on the serial monitoring of cardiac biomarkers. These studies suggest that transient, clinically non-relevant elevations in cardiac biomarkers can be observed after mRNA vaccine administrations both in healthy subjects and patients with pre-existing disorders, as observed in our cohort. One case of confirmed myopericarditis presenting with overt cardiac symptoms was reported in a study focusing on a demographically higher-risk group of 301 adolescents [64]. No additional clinically relevant events were recorded in independent cohorts of 777 [65], 324 [66], and 162 [67] subjects, despite 0.62–5% troponin elevation rates. Taken together, these data seem to indicate that the spectrum of transient clinical/pathological events ranging from the subclinical elevation of markers of cardiac injury after mRNA vaccine exposure to overt vaccine-related myocarditis might subtend unique pathogenic mechanisms, with only limited overlaps with virus-triggered and autoimmune myocarditis [68]. Enhanced susceptibility to oxidative stress (possibly exacerbated by disease-specific mechanisms in the setting of SLE and CTDs [67] and impaired regulation of IL-16/IL-18-biassed inflammatory responses [68,69] have been proposed as potential distinctive traits of vaccine-associated myocarditis and might account for troponin alterations uncoupled from clinically overt myocarditis relapses in the short- and long-term follow-up of patients receiving mRNA vaccines in the context of cardiac and non-cardiac autoimmunity. Confounders related to baseline systemic activity in these patients suggest the need for further translational studies in this setting.

Despite showing consistency with existing evidence in the literature, this work has some limitations that should be carefully considered for a correct interpretation of the data. First, the small size of our cohort prevents full generalisation of our findings, warranting further research in this field. Indeed, definite SLE-associated myocarditis is a rare condition [70], which inevitably reduces the ceiling of potential enrolment. Second, data collection was retrospective, which increases the risk of potentially missing minor clinical events that were not recorded in clinical charts. Third, the timing of laboratory data collection was not homogeneous, reflecting the complexity of real-life practice. Laboratory exams were also limited to routine testing. Therefore, serological response to vaccines was not assessed, and no conclusion about vaccine efficacy could be extrapolated. Furthermore, we could not ascertain whether the measurement of selected cytokines could have a role in patients with SLE and allied conditions to increase the sensitivity and specificity of myocarditis diagnosis.

Notwithstanding these limitations, our original findings add information concerning the long-term safety of SARS-CoV-2 vaccination in autoimmune patients with rare manifestations and can contribute to the management of these challenging cases.

## 5. Conclusions

Our work suggests that mRNA-based anti-COVID-19 vaccines are safe in patients with SLE and other lupus-like conditions with a history of myocarditis, both in the short and long term. Further studies and meta-analyses of aggregated data are needed to further validate our results.

## Figures and Tables

**Table 1:** Patients’ specific disease and baseline characteristics.

Feature	Value
SLE|UCTD|MCTD, N (%)	10 (83)|1 (8)|1 (8)
Sex (female), N (%)	7 (58)
Age (years), median (IQR)	48 (44–52)
Disease Duration (year), median (IQR)	17 (4–22)
Constitutional, N (%)	11 (92)
Mucocutaneous, N (%)	8 (67)
Neuropsychiatric, N (%)	3 (25)
Musculoskeletal, N (%)	9 (75)
Cardiological, N (%)	12 (100)
Gastroenterological, N (%)	3 (25)
Ophthalmologic, N (%)	1 (8)
Renal, N (%)	5 (42)
Haematologic, N (%)	8 (67)
Anti DNA	8 (67)
Anti-Sm	4 (33)
Anti-Ro	2 (17)
Anti-La	1 (8)
Anti-RNP	2 (17)
Anti-Cardiolipine	5 (42)
Anti-β2GPI	4 (33)
Lupus anticoagulant	2 (17)
Anti-PS/PT	2 (17)

SLE—Systemic Lupus Erythematosus; UCTD—Undifferentiated Connective Tissue Disease; MCTD—Mixed Connective Tissue Disease; IQR—interquartile range; anti-β2GPI—anti-β2-glicoprotein; anti-PS/PT—anti-Phosphatidylserine/Prothrombin.

**Table 2:** SLE features across different timepoints.

SLE Patients (10)	Before Vaccination	After Vaccination		
		Short-Term	Medium-Term	Long-Term
Patients in LLDAS: n (%)	8 (80)	8 (80)	8 (80)	10 (100)
Patients in DORIS: n (%)	7 (70)	7 (70)	8 (80)	9 (90)
SLEDAI-2k: median (IQR)	2 (0–3)	2 (0–4)	1 (0–2)	0 (0–2) *
BILAG Constitutional: median (IQR)	0 (0–0)	0 (0–1) ^#^	0 (0–0)	0 (0–0)
BILAG Mucocutaneous: median (IQR)	0 (0–1)	0 (0–0.75)	0 (0–0)	0 (0–0)
BILAG Neuropsychiatric: median (IQR)	0 (0–0)	0 (0–0)	0 (0–0)	0 (0–0)
BILAG Musculoskeletal: median (IQR)	0 (0–0)	0 (0–1)	0 (0–0)	0 (0–0)
BILAG Cardiological: median (IQR)	0 (0–0)	0 (0–0)	0 (0–0)	0 (0–0)
BILAG Gastroenterological: median (IQR)	0 (0–0)	0 (0–0)	0 (0–0)	0 (0–0)
BILAG Ophthalmologic: median (IQR)	0 (0–0)	0 (0–0)	0 (0–0)	0 (0–0)
BILAG RENAL: median (IQR)	0 (0–0)	0 (0–0)	0 (0–0)	0 (0–0)
BILAG Haematologic: median (IQR)	0 (0–1)	1 (0–1)	1 (0–1)	0 (0–1)
SLICC Damage Index: median (IQR)	1 (1–3)	2 (1–3)	2 (1–3)	2 (1–3)

* *p* < 0.05 compared to after vaccination by signed-rank test. ^#^ *p* < 0.05 compared to pre-vaccination by the Wilcoxon signed-rank test.

## Data Availability

The data presented in this study are available on request from the corresponding author. The data are not publicly available due to privacy restrictions.

## Supplementary Materials

The following supporting information can be downloaded at https://www.mdpi.com/article/10.3390/microorganisms13102266/s1, Table S1: Laboratory Exams at baseline and at long term evaluation.
