# Supplementary material for: Long-Term Safety of Anti-COVID-19 mRNA Vaccines in Patients with Systemic Lupus Erythematosus and Lupus-like Diseases with a Previous History of Myocarditis
Source: Microorganisms. 2025 Sep 26;13(10):2266. doi: 10.3390/microorganisms13102266 (PMC12566343; doi:10.3390/microorganisms13102266)
Supplement: Supplementary file 1 [file microorganisms-13-02266-s001.zip › microorganisms-3835350-supplementary.pdf]

Supplemental Materials

Long-Term Safety of Anti-COVID-19 mRNA Vaccines in Patients with Systemic Lupus Erythematosus and Lupus-like Diseases with a Previous History of Myocarditis

Giovanni Benanti [1], Marta Secci [1,2], Andrea Villatore [3,4], Sara Angiulli [1,3], Chiara Calabrese [1,3], Gabriele Domenico Gallina [1], Veronica Batani [1], Giacomo De Luca [1,3], Corrado Campochiaro [1,3], Giuseppe Pizzetti [5], Giovanni Peretto [3,4], Simone Sala [4], Enrica P. Bozzolo [1], Luca Moroni [1,3], Marco Matucci-Cerinic [1,3], Giuseppe A. Ramirez [1,3,*] and Lorenzo Dagna [1,3]

[1] Unit of Immunology, Rheumatology, Rheumatology, Allergy and Rare Diseases, IRCCS San Raffaele Scientific Institute, Via Olgettina 60, 20132 Milan, Italy
[2] Faculty of Medicine, University of Cagliari, Strada Provinciale 8, 09042 Monserrato, Italy
[3] Faculty of Medicine, Vita-Salute San Raffaele University, Via Olgettina 58, 20132 Milan, Italy
[4] Multidisciplinary Disease Unit for Myocarditis and Arrhythmogenic Cardiomyopathies, IRCCS San Raffaele Scientific Institute, Via Olgettina 60, 20132 Milan, Italy
[5] Unit of Cardiology, IRCCS San Raffaele Scientific Institute, Via Olgettina 60, 20132 Milan, Italy
* Correspondence: ramirez.giuseppealvise@hsr.it

Table S1 – Laboratory Exams at baseline and at long term evaluation

ITEM	BASELINE EVALUATION	LONG TERM EVALUATION
**Haemoglobin (g/l) Median (IQR)**	144 (132 - 151)	140 (137 - 150)
**White Blood Cells (cell/mm3) Median (IQR)**	5100 (4337.5-6300)	5000 (4600-6400)
**Neutrophil % Median (IQR)**	55.2 (51-60.6)	58 (54-62)
**Lymphocyte % Median (IQR)**	30.15 (27.1-34.25)	28.45 (25.5-33)
**Monocyte % Median (IQR)**	10 (7.8-10.65)	9 (7.8-11)
**Eosinophil % Median (IQR)**	2.2 (1-3.15)	2 (1.3-2.625)
**Basophil % Median (IQR)**	0.6 (0.4-1)	0.5 (0-1.025)
**Platelets (cell/mm3) Median (IQR)**	231 (197.75-260.5)	196 (176-254)
**Complement fraction C3 (g/l) Median (IQR)**	1.04 (0.9325-1.09)	1.3 (0.95-1.37)
**Complement fraction C4 (g/l) Median (IQR)**	0.175 (0.13-0.2)	0.23 (0.2-0.3)
**Anti-DNA titres**		
**Negative N (%)**	7 (58)	9 (75)
**Low N (%)**	1 (8)	2 (16)
**Moderate N (%)**	3 (25)	0 (0)
**High N (%)**	1 (8)	1 (8)
